# Supplementary material for: The Association of Insomnia with Febrile Neutropenia, Leucopenia, and Infection in Women Receiving Adjuvant Chemotherapy for Breast Cancer
Source: Cancers (Basel). 2025 May 30;17(11):1838. doi: 10.3390/cancers17111838 (PMC12153840; doi:10.3390/cancers17111838)
Supplement: Supplementary file 1 [file cancers-17-01838-s001.zip › Table S1.pdf]

Table S1: Baseline patients' characteristics

Characteristics	Overall (N=2,104)	QOL subgroup (N=1,731)	Chemotherapy regimen (N=1,731)		
			CEF (N=584)	EC/T (N=576)	AC/T (N=571)
**Age**					
Median	47.7	47.6	48.1	47.5	47.4
**Performance Status**					
0	1766 (84.0%)	1453 (83.9%)	491 (84.1%)	481 (83.5%)	481 (84.2%)
1	331 (15.7%)	274 (15.8%)	91 (15.6%)	95 (16.5%)	88 (15.4%)
2	6 (0.3%)	4 (0.2%)	2 (0.3%)	0 (%)	2 (0.4%)
**Menopausal Status**					
Pre	1433 (68.1%)	1201 (69.4%)	405 (69.4%)	407 (70.7%)	389 (68.1%)
Post	670 (31.9%)	530 (30.6%)	179 (30.7%)	169 (29.3%)	182 (31.9%)
**Race**					
White	1866 (88.7%)	1565 (90.4%)	537 (92.0%)	527 (91.5%)	501 (87.7%)
Black	98 (4.7%)	59 (3.4%)	14 (2.4%)	21 (3.7%)	24 (4.2%)
Haiwaiian or Pacific Islander	2 (0.1%)	0 (%)	0 (%)	0 (%)	0 (%)
Asian	74 (3.5%)	55 (3.2%)	8 (1.4%)	20 (3.5%)	27 (4.7%)
Aboriginal	20 (1.0%)	16 (0.9%)	8 (1.4%)	5 (0.9%)	3 (0.5%)
Unknown	43 (2.0%)	36 (2.1%)	17 (2.9%)	3 (0.5%)	16 (2.8%)
**T Stage**					
1	738 (35.1%)	596 (34.4%)	194 (33.2%)	198 (34.4%)	204 (35.7%)
2	1145 (54.5%)	948 (54.8%)	325 (55.7%)	308 (53.5%)	315 (55.2%)
3	188 (8.9%)	161 (9.3%)	57 (9.8%)	55 (9.6%)	49 (8.6%)
4	27 (1.3%)	21 (1.2%)	8 (1.4%)	10 (1.7%)	3 (0.5%)
X	5 (0.2%)	5 (0.3%)	(%)	5 (0.9%)	(%)
**N Stage**					
0	588 (28.0%)	455 (26.3%)	151 (25.9%)	153 (26.6%)	151 (26.4%)
1	1362 (64.7%)	1158 (66.9%)	389 (66.6%)	384 (66.6%)	385 (67.4%)
2	153 (7.3%)	118 (6.8%)	44 (7.5%)	39 (6.8%)	35 (6.1%)

Legend: Overall: All patients included in the MA.21 trial, QOL: Quality of life, QOL subgroup: All patients for whom QOL data were available. CEF: Cyclophosphamide + Epirubicin+ Fluorouracil, EC/T: Epirubicin + Cyclophosphamide, followed by paclitaxel, AC/T: Doxorubicine + Cyclophosphamide, followed by Paclitaxel. All numbers were rounded to one decimal. Missing data for each characteristic were ≤0.1% and are not reported in the table.
