# Supplementary material for: The Association of Insomnia with Febrile Neutropenia, Leucopenia, and Infection in Women Receiving Adjuvant Chemotherapy for Breast Cancer
Source: Cancers (Basel). 2025 May 30;17(11):1838. doi: 10.3390/cancers17111838 (PMC12153840; doi:10.3390/cancers17111838)
Supplement: Supplementary file 1 [file cancers-17-01838-s001.zip › Table S2.pdf]

Table S2: Distribution of insomnia and QOL data

Characteristics	QOL subgroup (N=1,731)$	CEF (N=584)	EC/T (N=576)	AC/T (N=571)
**EORTC global QOL at baseline**				
N	1720	582	570	568
Median	83.3	83.3	83.3	83.3
p		0.34		
**EORTC Q 11: sleep problem (score distribution)**#				
1	531 (30.7%)	182 (31.2%)	182 (31.6%)	167 (29.3%)
2	782 (45.2%)	268 (45.9%)	248 (43.1%)	266 (46.6%)
3	268 (15.5%)	85 (14.6%)	100 (17.4%)	83 (14.5%)
4	138 (7.8%)	44 (7.5%)	42 (7.3%)	52 (9.1%)
Missing	12 (0.7%)	5 (0.9%)	4 (0.7%)	3 (0.5%)
p	P=0.73			
**EORTC Q 11: "insomnia" = score ≥ 3 (%)** #				
No	1313 (75.9%)	450 (77.1%)	430 (74.7%)	433 (75.8%)
Yes	406 (23.5%)	129 (22.1%)	142 (24.7%)	135 (23.6%)
Missing	12 (0.7%)	5 (0.9%)	4 (0.7%)	3 (0.5%)
p		0.83		
**EORTC Emotional Function domain: % with score ≤70** #				
No	906 (52.3%)	309 (52.9%)	293 (50.9%)	304 (53.2%)
Yes	824 (47.6%)	275 (47.1%)	283 (49.1%)	266 (46.6%)
Missing	1 (0.1%)	0 (0%)	0 (0%)	1 (0.2%)
p		0.67		

Legend: QOL: Quality of life sample, which include all patients for whom QOL data were available, EORTC: European Organization for the Research and Treatment of Cancer, CEF: Cyclophosphamide + Epirubicin+ Fluorouracil, EC/T: Epirubicin + Cyclophosphamide, followed by paclitaxel, AC/T: Doxorubicine + Cyclophosphamide, followed by Paclitaxel. All percentages were rounded to one decimal.

$ 1,720 patients completed the EORTC QLQ-C30

Using the worst score at either baseline or follow-up 1 to 4.
