# Supplementary material for: The Association of Insomnia with Febrile Neutropenia, Leucopenia, and Infection in Women Receiving Adjuvant Chemotherapy for Breast Cancer
Source: Cancers (Basel). 2025 May 30;17(11):1838. doi: 10.3390/cancers17111838 (PMC12153840; doi:10.3390/cancers17111838)
Supplement: Supplementary file 1 [file cancers-17-01838-s001.zip › Table S3.pdf]

Table S3. Outcomes and some covariates in the QOL sample and by chemotherapy regimen

Outcomes	QOL sample (N=1731)	CEF (n=584)	EC/T (n=576)	AC/T (n=571)
**Febrile neutropenia**				
No (Grades 0-2)	1482 (85.6%)	457 (78.2%)	482 (83.7%)	543 (95.1%)
Yes (Grades 3-5)	249 (14.4%)	127 (21.8%)	94 (16.3%)	28 (4.9%)
p		<0.0001		
**Leucopenia**				
Grade 0	298 (17.2%)	57 (9.8%)	78 (13.5%)	163 (28.6%)
Grade 1	273 (15.8%)	61 (10.5%)	71 (12.3%)	141 (24.7%)
Grade 2	370 (21.4%)	138 (23.6%)	100 (17.4%)	132 (23.1%)
Grade 3	387 (22.4%)	150 (25.7%)	125 (21.7%)	112 (19.6%)
Grade 4	403 (23.3%)	178 (30.5%)	202 (35.1%)	23 (4.0%)
Grade 5	0 (0%)	0 (0%)	0 (0%)	0 (0%)
p		<0.0001		
**Infection**				
Grade 0	1183 (68.3%)	396 (67.8%)	357 (62.0%)	430 (75.3%)
Grade 1	130 (7.5%)	46 (7.9%)	44 (7.6%)	40 (7.0%)
Grade 2	268 (15.5%)	81 (13.9%)	110 (19.1%)	77 (13.5%)
Grade 3	148 (8.6%)	61 (10.5%)	63 (10.9%)	24 (4.2%)
Grade 4	2 (0.1%)	0 (0%)	2 (0.4%)	0 (0%)
Grade 5	0 (0%)	0 (0%)	0 (0%)	0 (0%)
p		<0.0001		
**Chemotherapy delay**				
No	510 (29.5%)	182 (31.2%)	140 (24.3%)	188 (32.9%)
Yes	1221 (70.5%)	402 (68.4%)	436 (75.7%)	383 (67.1%)
p		0.003		
**Chemotherapy dose reduction**				
No	1272 (73.5%)	364 (62.3%)	374 (64.9%)	534 (93.5%)
Yes	459 (26.5%)	220 (37.7%)	202 (35.1%)	37 (6.5%)
p		<0.0001		
**Use of prophylactic antibiotics**				
No	1029 (59.5%)	9 (1.5%)	488 (84.7%)	532 (93.2%)
Yes	702 (40.5%)	575 (98.5%)	88 (15.3%)	39 (6.8%)
p		<0.0001		

**Use of G-CSF**				
No	826 (47.7%)	342 (58.6%)	3 (0.5%)	481 (84.2%)
Yes	905 (52.3%)	242 (41.4%)	573 (99.5%)	90 (15.8%)
P value		<0.0001		
**Use of corticosteroids**				
No	1659 (95.8%)	564 (96.6%)	546 (94.8%)	549 (96.1%)
Yes	72 (4.2%)	20 (3.4%)	30 (5.2%)	22 (3.9%)
p		0.28		

Legend: QOL: Quality of life sample, which include all patients for whom QOL data were available, CEF: Cyclophosphamide + Epirubicin+ Fluorouracil, EC/T: Epirubicin + Cyclophosphamide, followed by paclitaxel, AC/T: Doxorubicine + Cyclophosphamide, followed by Paclitaxel, G-CSF: Granulocyte- colony stimulating factor. All percentages were rounded to one decimal.
