# Supplementary material for: The Association of Insomnia with Febrile Neutropenia, Leucopenia, and Infection in Women Receiving Adjuvant Chemotherapy for Breast Cancer
Source: Cancers (Basel). 2025 May 30;17(11):1838. doi: 10.3390/cancers17111838 (PMC12153840; doi:10.3390/cancers17111838)
Supplement: Supplementary file 1 [file cancers-17-01838-s001.zip › Table S4.pdf]

Table S4: Multivariate analyses for leucopenia using the EORTC QLQ-C30 questionnaire

Predictor	Multivariate analysis		Second multivariate analysis excluding emotional functioning score	
	Odds Ratio (95% CI)	p	Odds Ratio (95%CI)	p
Insomnia (Yes vs. No)	0.97 (0.74-1.28)	0.82	0.95 (0.73-1.24)	0.69
G-CSF (Yes vs. No)	6.73 (3.70-12.24)	<0.0001	6.76 (3.72-12.30)	<0.0001
Prophylactic antibiotics (Yes vs. No)	4.38 (2.11-9.11)	<0.0001	4.41 (2.12-9.18)	<0.0001
Age	1.00 (0.97-1.02)	0.67	1.00 (0.97-1.02)	0.71
Race (Aboriginal vs. Caucasian)	0.81 (0.22-2.95)	0.74	0.81 (0.22-2.97)	0.75
Race (Asian vs. Caucasian)	1.22 (0.59-2.53)	0.60	1.22 (0.59-2.53)	0.60
Race (Black vs. Caucasian)	1.06 (0.49-2.29)	0.88	1.06 (0.49-2.28)	0.88
Race (Unknown vs. Caucasian)	0.57 (0.26-1.28)	0.17	0.57 (0.25-1.27)	0.17
Treatment Arm (CEF vs. AC/T)	0.71 (0.33-1.52)	0.37	0.70 (0.32-1.51)	0.37
Treatment Arm (EC/T vs. AC/T)	0.43 (0.22-0.81)	0.01	0.42 (0.22-0.80)	0.01
Menopausal Status (Post vs. Pre)	1.05 (0.72-1.54)	0.79	1.04 (0.71-1.52)	0.83
Performance Status (1+ vs. 0)	1.18 (0.82-1.70)	0.38	1.17 (0.81-1.68)	0.40
N Stage (1 vs. 0)	0.97 (0.71-1.32)	0.86	0.97 (0.72-1.32)	0.85
N Stage (2 vs. 0)	1.03 (0.57-1.87)	0.92	1.02 (0.56-1.86)	0.94
T Stage (2 vs. 1)	0.96 (0.72-1.28)	0.80	0.97 (0.72-1.28)	0.80
T Stage (3+ vs. 1)	1.05 (0.66-1.67)	0.85	1.05 (0.66-1.67)	0.85
Emotional Functioning score	1.00 (1.00-1.01)	0.53	NA	NA

Legend: CEF: Cyclophosphamide + Epirubicin+ Fluorouracil, EC/T: Epirubicin + Cyclophosphamide, followed by paclitaxel, AC/T: Doxorubicine + Cyclophosphamide, followed by Paclitaxel, G-CSF : Granulocyte colony stimulating factor, All numbers were rounded to two decimals. Insomnia defined using EORTC criteria (Q 11 score ≥3).
