# Supplementary material for: The Association of Insomnia with Febrile Neutropenia, Leucopenia, and Infection in Women Receiving Adjuvant Chemotherapy for Breast Cancer
Source: Cancers (Basel). 2025 May 30;17(11):1838. doi: 10.3390/cancers17111838 (PMC12153840; doi:10.3390/cancers17111838)
Supplement: Supplementary file 1 [file cancers-17-01838-s001.zip › Table S5.pdf]

Table S5: Multivariate analysis for infections using the EORTC QLQ-C30 questionnaire

Predictor	Multivariate analysis		Second multivariate analysis excluding emotional functioning score	
	Odds Ratio (95% CI)	p	Odds Ratio (95%CI)	p
Insomnia (Yes vs. No)	1.13 (0.91-1.40)	0.27	1.14 (0.92-1.40)	0.23
G-CSF (Yes vs. No)	1.29 (0.97-1.73)	0.08	1.31 (0.98-1.75)	0.07
Prophylactic antibiotics (Yes vs. No)	1.39 (0.95-2.03)	0.09	1.39 (0.95-2.02)	0.09
Age	0.98 (0.97-1.00)	0.07	0.98 (0.97-1.00)	0.07
Race (Aboriginal vs. Caucasian)	1.62 (0.59-4.44)	0.35	1.62 (0.59-4.44)	0.35
Race (Asian vs. Caucasian)	0.39 (0.18-0.83)	0.02	0.39 (0.18-0.83)	0.02
Race (Black vs. Caucasian)	0.50 (0.26-0.96)	0.04	0.50 (0.26-0.95)	0.04
Race (Unknown vs. Caucasian)	1.12 (0.55-2.29)	0.76	1.12 (0.55-2.28)	0.76
Treatment Arm (CEF vs. AC/T)	0.97 (0.63-1.50)	0.90	0.97 (0.63-1.49)	0.88
Treatment Arm (EC/T vs. AC/T)	1.45 (1.02-2.06)	0.04	1.43 (1.00-2.02)	0.05
Menopausal Status (Post vs. Pre)	1.02 (0.76-1.37)	0.90	1.02 (0.76-1.37)	0.91
Performance Status (1+ vs. 0)	1.35 (1.03-1.78)	0.03	1.35 (1.03-1.78)	0.03
N Stage (1 vs. 0)	0.92 (0.73-1.17)	0.49	0.92 (0.73-1.17)	0.51
N Stage (2 vs. 0)	1.15 (0.74-1.78)	0.54	1.15 (0.74-1.78)	0.54
T Stage (2 vs. 1)	1.03 (0.82-1.29)	0.83	1.03 (0.82-1.29)	0.80
T Stage (3+ vs. 1)	0.96 (0.66-1.38)	0.81	0.96 (0.66-1.38)	0.81
Emotional Functioning score	1.00 (1.00-1.01)	0.95	NA	NA

Legend:CEF: Cyclophosphamide + Epirubicin+ Fluorouracil, EC/T: Epirubicin + Cyclophosphamide, followed by paclitaxel, AC/T: Doxorubicine + Cyclophosphamide, followed by Paclitaxel, G-CSF : Granulocyte colony stimulating factor, All numbers were rounded to two decimals. Insomnia defined using EORTC criteria (Q 11 score ≥3).
