# Supplementary material for: The Association of Insomnia with Febrile Neutropenia, Leucopenia, and Infection in Women Receiving Adjuvant Chemotherapy for Breast Cancer
Source: Cancers (Basel). 2025 May 30;17(11):1838. doi: 10.3390/cancers17111838 (PMC12153840; doi:10.3390/cancers17111838)
Supplement: Supplementary file 1 [file cancers-17-01838-s001.zip › Table S6.pdf]

Table S6: Multivariate analyses for chemotherapy dose reductions

Predictor	Multivariate analysis		Second multivariate analysis excluding Emotional functioning score	
	Odds Ratio (95% CI)	p	Odds Ratio (95%CI)	p
Insomnia (Yes vs. No)	1.67 (1.30-2.15)	<0.0001	1.67 (1.31-2.13)	<0.0001
G-CSF (Yes vs. No)	3.43 (2.49-4.74)	<0.0001	3.42 (2.48-4.73)	<0.0001
Prophylactic antibiotics (Yes vs. No)	1.75 (1.16-2.66)	0.01	1.76 (1.16-2.66)	0.01
Age	1.05 (1.02-1.07)	<0.0001	1.05 (1.02-1.07)	<0.0001
Race (Aboriginal vs. Caucasian)	0.85 (0.23-3.07)	0.80	0.85 (0.23-3.07)	0.80
Race (Asian vs. Caucasian)	1.37 (0.67-2.81)	0.39	1.37 (0.67-2.82)	0.39
Race (Black vs. Caucasian)	0.81 (0.41-1.61)	0.55	0.82 (0.41-1.62)	0.56
Race (Unknown vs. Caucasian)	0.61 (0.22-1.66)	0.33	0.61 (0.22-1.66)	0.33
Treatment Arm (CEF vs. AC/T)	4.39 (2.59-7.42)	<0.0001	4.40 (2.60-7.45)	<0.0001
Treatment Arm (EC/T vs. AC/T)	3.10 (2.00-4.80)	<0.0001	3.12 (2.01-4.83)	<0.0001
Menopausal Status (Post vs. Pre)	1.04 (0.75-1.44)	0.83	1.04 (0.75-1.44)	0.82
Performance Status (1+ vs. 0)	1.09 (0.79-1.49)	0.62	1.09 (0.79-1.49)	0.61
N Stage (1 vs. 0)	0.94 (0.71-1.23)	0.64	0.94 (0.71-1.23)	0.64
N Stage (2 vs. 0)	1.12 (0.68-1.84)	0.64	1.12 (0.68-1.85)	0.64
T Stage (2 vs. 1)	1.21 (0.93-1.56)	0.16	1.20 (0.93-1.56)	0.16
T Stage (3+ vs. 1)	1.13 (0.75-1.71)	0.56	1.13 (0.75-1.71)	0.56
Emotional Functioning Domain	1.00 (0.99-1.01)	0.97	NA	NA

Legend: CEF: Cyclophosphamide + Epirubicin+ Fluorouracil, EC/T: Epirubicin + Cyclophosphamide, followed by paclitaxel, AC/T: Doxorubicine + Cyclophosphamide, followed by Paclitaxel, G-CSF : Granulocyte colony stimulating factor, All numbers were rounded to two decimals. Insomnia defined using EORTC criteria (Q 11 score ≥3).
