# Supplementary material for: The Association of Insomnia with Febrile Neutropenia, Leucopenia, and Infection in Women Receiving Adjuvant Chemotherapy for Breast Cancer
Source: Cancers (Basel). 2025 May 30;17(11):1838. doi: 10.3390/cancers17111838 (PMC12153840; doi:10.3390/cancers17111838)
Supplement: Supplementary file 1 [file cancers-17-01838-s001.zip › Table S7.pdf]

Table S7: Dose-response analyses using the EORTC-QLQ C30 insomnia definition.

Febrile neutropenia according to the number of questionnaires meeting the definition of insomnia with the EORTC QLQ-C30:	No (n=1477)	Yes (n=254)	Total n=1731
0	47.39%	38.58%	
1	23.22%	24.41%	
2	13.68%	16.54%	
3	9.00%	9.45%	
4	4.87%	7.48%	
5	1.83%	3.54%	
			p=0.0524 Chi square<br>ANOVA 0.0149
Delay in chemotherapy (yes) - according to the number of questionnaires meeting the definition of insomnia with the EORTC QLQ-C30:	No (n= 510)	Yes (n=1221)	Total n=1731
0	46.86%	45.78%	
1	24.90%	22.77%	
2	13.33%	14.41%	
3	8.04%	9.50%	
4	6.08%	4.91%	
5	0.78%	2.62%	
			p=0.1217 (Chi square)<br>ANOVA p=0.9071
Chemotherapy dose reduction according to the number of questionnaires meeting the definition of insomnia with the EORTC QLQ-C30:	No (n=1272)	Yes (n=459)	Total n =1731
0	49.06%	37.91%	
1	22.72%	25.27%	
2	13.21%	16.56%	
3	8.49%	10.68%	
4	4.72%	6.75%	
5	1.81%	2.82%	

			p=0.0020 Chi square<br>ANOVA p = 0.0005
Febrile neutropenia according to the presence or not of insomnia at baseline (according the definition of insomnia with the EORTC QLQ-C30):	No (n=1392)	Yes (n=230)	
No (0)	77.73%	71.30%	
Yes (1)	22.27%	28.70%	
			p=0.0324 (Chi square)
Delay in chemotherapy according to the presence or not of insomnia at baseline (according the definition of insomnia with the EORTC QLQ-C30):	No (n=474)	Yes (n=1148)	Total (n=1622)
No (0)	79.32%	75.78%	
Yes (1)	20.68%	24.22%	
			p=0.1243 (Chi Square)
Chemotherapy dose reduction according to the presence or not of insomnia at baseline (according the definition of insomnia with the EORTC QLQ-C30):	No (n=1198)	Yes (n=424)	Total (n=1622)
No (0)	78.13%	73.11%	
Yes (1)	21.87%	26.89%	
			P=0.0354 (Chi square)
